# IgM has a better relative distribution in inflammation sites and tumor tissues than IgG

**DOI:** 10.1186/s12951-025-03213-4

**Published:** 2025-03-28

**Authors:** Abdulaziz M. Aldayel, Mohammad Bosaeed, Sarah Almansour, Naif Khalaf Alharbi, Mohammed Alenazi, Haya A. Aljami, Omar Aldibasi, Abdulrhman Aljouie, Haiyue Xu, Zhengrong Cui

**Affiliations:** 1 College of Pharmacy, Division of Molecular Pharmaceutics and Drug Delivery, The University of Texas at Austin, Austin, TX 78712 USA; 2 Nanomedicine Department, King Abdullah International Medical Research Center (KAIMRC), King Abdulaziz Medical City (KAMC), 11426 Riyadh, Saudi Arabia; 3 Infectious Diseases Research Department, King Abdullah International Medical Research Center (KAIMRC), King Abdulaziz Medical City (KAMC), 11426 Riyadh, Saudi Arabia; 4 Department of Medicine, King Abdulaziz Medical City (KAMC), 11426 Riyadh, Saudi Arabia; 5 Department of Biostatistics and Bioinformatics, King Abdullah International Medical Research Center (KAIMRC), King Saud Bin Abdulaziz University for Health Sciences, 11426 Riyadh, Saudi Arabia; 6 College of Pharmacy & College of Medicine, King Saud Bin Abdulaziz University for Health Sciences (KSAU-HS), King Abdulaziz Medical City (KAMC), 11426 Riyadh, Saudi Arabia

**Keywords:** Antibodies, Nanoparticle size, Distribution, Inflammation, Tumor

## Abstract

Immunoglobulins (Igs) play a crucial role in host’s defense and in developing therapies against inflammatory diseases and cancer. Herein, we first studied the relative distribution of IgM and IgG in mouse models with acute or chronic inflammation. We found that IgM showed a more selective distribution towards inflammation sites than IgG. Similarly, in a tumor-bearing mouse model, IgM showed a higher tumor-to-blood or -to healthy organs ratio than IgG. We hypothesized that the difference in the sizes between IgM and IgG may have contributed to the differences in their relative distribution, which was supported by using an IgG nanoparticle system that was similar to IgM in size. To confirm the findings in clinics, we investigated IgM and IgG levels in the blood and bronchoalveolar lavage fluid (BALF) of patients diagnosed with fungal pneumonia and showed that the relative distribution of IgM was significantly higher than IgG in the BALF samples as compared to that in serum. Such an understanding of our immune system at the nano-level may help us develop more effective biotechnological interventions against inflammatory diseases and cancers.

**Graphical Abstract:**

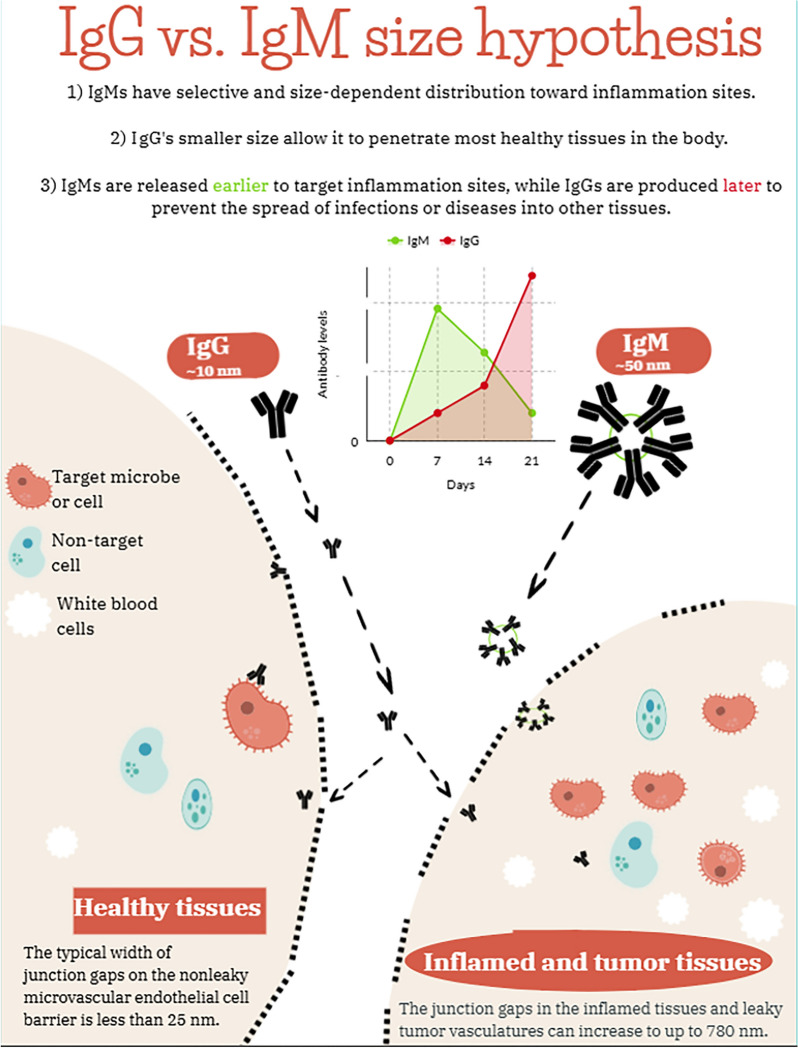

**Supplementary Information:**

The online version contains supplementary material available at 10.1186/s12951-025-03213-4.

## Introduction 

Natural antibodies are produced by plasma cells in response to pathogens or infected cells. IgM is the immunoglobulin produced before class switching to other Ig isotypes such as IgG [1–6]. Igs are an essential part of immunity and contribute to health and disease (e.g., host defense, inflammation, cancer, autoimmunity, etc.) [1–6]. IgM can be detected in the first week after the onset of illness or infection. IgG class switching occurs about 14 days after the onset of the illness [1–6]. IgG isotype switching involves a replacement of heavy chain constant regions from μ and δ to γ, which have enhanced affinity to pathogens, as compared to IgM [1–6]. IgM has multipoint binding sites to pathogens that confer high overall avidity with, however, relatively lower affinity to pathogens [1–6].

Another major difference between the two classes of antibodies is their molecular weights, and thus (particle) sizes, which affect their distributions in the body. IgMs are pentamers that are large (~ 50–100 nm), mainly found in the blood circulation and, to a lesser extent, the lymph nodes [1–6]. Unlike IgMs, the high-affinity IgGs are much smaller in size (~ 5–10 nm), which allows them to passively diffuse out of the blood circulation into tissues, even crossing the placental barrier to provide immunity to the fetus [1, 2]. It is noted that other active mechanisms of IgG crossing to healthy tissues have been reported, e.g. IgG can also cross to the fetus via the neonatal Fc receptor (FcRn) in the placenta [1, 2]. IgGs have a significant function in long-term immunity, but natural and adaptive IgMs are effective in the clearance of cancer and apoptotic cells, modulation of inflammatory responses to pathogens, and regulation of autoimmune diseases [1–6]. These differences have been exploited in pre-clinical and clinical studies [7–9]. For example, Subramanian et al. showed that IgMs have a higher accumulation in intramuscular acute inflammation sites induced by *E. coli* infection than in healthy muscles in a rat model, as compared to IgGs [7]. The ratios of IgM in infected muscles vs. healthy muscles or blood were higher at several time points (i.e. 4, 8, 16, 24 and 36 h) post-injection as compared to IgG. At 24 h, the IgM ratio in infected muscles vs. healthy muscles was 2.4 times higher than IgG ratio, marking the maximum reported difference in IgM to IgG ratio towards the infected muscles [7]. As a possible explanation, the authors suggested that the IgM used in their study (IgM 16.88) had a higher avidity towards dead lymphocytes and granulocytes at the inflammation sites, than the live cells at the healthy tissues [7]. In clinical studies, it was also reported that IgM levels are significantly increased in several malignant and benign tumors in mammary tissues, relative to in non-tumoral mammary tissues [8, 9]. The IgG levels in the same tumor tissues were not significantly different from non-tumoral mammary tissues [8, 9]. Similarly, several studies suggested that the high avidity of IgM towards apophatic cells is possibly the reason for the higher distribution and/or accumulation of IgM in tumors and inflammation sites [10–12]. Another potential explanation that has not been well explored is the particle size difference between IgM and IgG. Endothelial gap junctions in non-leaky healthy tissues tend to be less than 25 nm, whereas the microvascular gap junction in leakyand diseased tissues can be up to 780 nm in size [13].

We have recently studied the effect of nanoparticle size on their distribution in chronic inflammation sites [14]. Larger nanoparticles of 100–200 nm in hydrodynamic diameter tend to have a more selective distribution in chronic inflammation sites than smaller nanoparticles of 10–20 nm [14], which we attributed to the extravasation through leaky vasculature and subsequent inflammatory cell-mediated sequestration (ELVIS) phenomenon [14–19]. The blood vessels in tumor tissues are also leaky and the enhanced permeability and retention (EPR) effect is used to explain the increased distribution of nanoparticles, including IgG antibodies, in tumor tissues [20–23]. In the present study, we hypothesized that after being injected intravenously, IgM antibodies will show more selective distribution and accumulation in inflammation sites and tumor tissues than IgG antibodies. We tested the hypothesis using fluorescently labeled non-binding IgG and IgM antibodies in mouse models with acute or chronic inflammations or with orthotopic mammary tumors. Moreover, we measured human IgM and IgG levels in the plasma and BALF samples of patients with fungal pneumonia. Our data showed the effect of fungal infection-induced lung inflammation on the distribution of IgG and IgM in BALF samples across the capillary wall and the alveolus wall into the alveolar fluid. There is evidence that the pulmonary capillaries surrounding the alveolus are leaky in infected and inflamed lungs [24–28].

## Methods

### Materials

N,N-dimethyl-9,9-biacridinium dinitrate (lucigenin), lipopolysaccharides (LPS) from *Salmonella enterica* serotype enteritidis, 3-aminophthalhydrazide, 5-amino-2,3-dihydro-1,4-phthalazinedione (luminol sodium salt), 3,3′-dithiobis(sulfosuccinimidyl propionate) (DTSSP) were from Sigma-Aldrich (St. Louis, MO). Normal mouse IgG Alexa Fluor^®^ 647 and normal mouse IgM Alexa Fluor^®^ 647 were from Santa Cruz Biotechnology (Dallas, TX). Human IgM ELISA Kits and Human IgG ELISA Kits were from ThermoFisher Scientific (Waltham, MA).

### Clinical study and data

The Institutional Review Board of the King Abdulaziz Medical City (KAMC) reviewed and approved the study (N. NRC22R/042/01) under the ethical standards of the responsible committees on human experimentation and the Declaration of Helsinki. Informed consent was obtained for participation in this study. Blood and BALF samples from 20 patients diagnosed with invasive pulmonary Aspergillus were collected and analyzed using ELISA. The commercial Human IgM ELISA Kits and Human IgG ELISA Kits were used following the manufacturer’s instructions. Blood and BALF samples were collected in plain tubes on different days post-diagnosis. All patients in this study have a second serious indication, which requires the administration of immunosupressents. Other medical history and pathological tests (i.e., age, gender, malignancy type, stem cell transplant recipient type, solid organ transplant recipient type, chronic lung disease type, nodules, consolidation, pleural effusion, halo sign, ground glass opacity, cavitary lesion, serum galactomannan test, BAL galactomannan test, BAL culture result, symptoms onset date, hypoxia, ICU admission, intubation, mortality, ESR test, CRP test, respiratory multiplex, and other alternative diagnoses) were also collected and analyzed. Samples were centrifuged for 5 min at 1500 ×*g* at room temperature, and serum was isolated and stored in cryo-tubes at − 80 °C until the day of analysis.

### Development of LPS-induced mouse models of acute and chronic inflammation.

All animal studies were approved by the Institutional Animal Care and Use Committee at The University of Texas at Austin. Female C57BL/6 mice (6–8 weeks) were from Charles River Laboratories (Wilmington, MA). For imaging, mice were fed with an alfalfa-free diet (Harlan, Indiana) to minimize unwanted background signals. An LPS-induced mouse model of chronic inflammation was established as previously described [29]. Briefly, LPS was dissolved in sterile PBS (pH 7.4, 10 mM) at a concentration of 1 mg/ml. On day 0, 50 μl of the solution was injected into the right rear footpad of each mouse. Acute inflammation was confirmed on day 3 using an IVIS^®^ Spectrum (Caliper, Hopkinton, MA) with a bioluminescence imaging system 20 min following intraperitoneal (i.p.) injection of luminol (100 mg/kg) (exposure time 60 s, large binning, field B). For the chronic inflammation studies, chronic inflammation was confirmed on day 8 using an IVIS^®^ Spectrum with a bioluminescence imaging system 20 min following i.p. injection of lucigenin (15 mg/kg) (exposure time 60 s, large binning, field B). Only mice that showed significant acute or chronic inflammation in the right foot were used (Supp. Figure 1).

### Kinetics of IgG and IgM in a mouse model of LPS-induced acute and chronic inflammation

Upon the confirmation of acute inflammation in the right rear foot (on day 3 post-LPS injection), mice were randomized to groups and injected intravenously (i.v.) with PBS or fluorescently labeled non-binding IgG or IgM (IgM Alexa Fluor^®^ 647, 40 μg/kg; IgG Alexa Fluor^®^ 647, 20 μg/kg to account for difference in fluorescence intensities). Mice were imaged using the IVIS^®^ Spectrum 3, 6, 12, and 24 h after the injection. All fluorescent units are in photons per second per centimeter square per steradian (p/s/cm^2^/sr). Data were analyzed using PK Solver [30]. Similarly, groups of mice with chronic inflammation in the right rear foot were i.v. injected (on day 9 post-LPS injection) with PBS or fluorescently labeled IgG or IgM (IgM Alexa Fluor^®^ 647, 40 μg/kg; IgG Alexa Fluor^®^ 647, 20 μg/kg to account for difference in fluorescence intensities). Mice were imaged using an IVIS^®^ Spectrum 3, 6, 12, 24 h and 48 h after the injection. Data were analyzed using PK Solver [30]. Fluorescence background from PBS mice was subtracted from the measured values to account for autofluorescence.

### Biodistribution of IgG and IgM in in a mouse model with orthotopic mammary tumors

MMTV-M-Wnt-1 (M-Wnt) mammary tumor cells (basal-like, triple-negative, claudin-low) were cloned from spontaneous mammary tumors in MMTV-Wnt-1 transgenic mice in a congenic C57BL/6 background [23, 31]. M-Wnt cells were cultured in RPMI 1640 medium at 37 °C, 5% CO_2_. The medium was supplemented with 10% fetal bovine serum (FBS), 100 U/mL of penicillin, and 100 μg/mL of streptomycin. All cell culture medium and reagents were from Invitrogen (Carlsbad, CA). M-Wnt tumors were established in female C57BL/6 mice (6–8 weeks, Charles River) by injecting M-Wnt tumor cells (5 × 10^5^ cells/mouse) subcutaneously in the ninth mammary fat pad of the mice. When tumors reached 6–9 mm in diameter, mice were i.v. injected with PBS, non-binding IgG or IgM. Both IgG and IgM were fluorescently labeled with Alexa Fluor^®^ 647. The dose of IgM was 40 µg/kg, 20 µg/kg for IgG so that the fluorescence intensities of the two antibodies injected in each mouse were identical. Mice were euthanized 24 h later to collect blood, tumor, and major organs (e.g., heart, kidneys, liver, spleen, and lung, gastrointestinal tract). All samples were then imaged using an IVIS Spectrum (Em/Ex of 465/600 nm). For blood samples, approximately 20 µl of blood per mouse was diluted in 500 µl of PBS to measure fluorescence intensity. The values for the PBS group’s organs and blood were subtracted from the measured values to account for autofluorescence.

### Preparation and characterization of redox-sensitive IgG nanoparticles (IgG-NPs)

Redox-sensitive IgG nanoparticles were prepared as previously described [16]. Briefly, normal mouse IgG Alexa Fluor^®^ 647 from Santa Cruz Biotechnology was diluted to 100 µg/ml in a 10 mM sodium chloride solution, pH 9.0. Aliquots (1.0 ml) of the IgG solution were transformed into nanoparticles by dropwise addition of 4.0 ml of a desolvating agent (i.e., ethanol/methanol, 50%/50%) under stirring (500 rpm) at room temperature. After the desolvation process, 100 μl of a DTSSP in water solution (i.e., 0.004%) were added and the mixture was incubated at room temperature for 24 h under stirring to induce particle crosslinking. Particle size was measured using a Malvern Nano ZS and morphology examined using transmission electron microscopy.

### Biodistribution of IgG and IgG-NPs in in a mouse model with orthotopic mammary tumors

M-Wnt tumors were established as described above in mice. When tumors reached 6–9 mm in diameter, mice were i.v. injected with PBS, IgG, or IgG-NPs. The IgG was fluorescently labeled with Alexa Fluor^®^ 647. The dose of IgG and IgG-NPs was about 20 µg IgG/kg, and the fluorescence intensities of the two samples were identical. Mice were euthanized 24 h later to collect blood, tumor, and major organs (except for the gastrointestinal tract (GI)). All samples were then imaged using an IVIS Spectrum (Em/Ex of 465/600 nm). For blood samples, approximately 20 µl of blood per mouse was diluted in 500 µl of PBS to measure fluorescence intensity. The values for the PBS group’s organs and blood were subtracted from the measured values to account for autofluorescence.

### Statistical analysis

Statistical analyses were completed by performing a fixed effects model and Tukey–Kramer adjustment for the kinetic study of IgG and IgM in mice with acute and chronic inflammation (Supp Table. S1). Wilcoxon two-sample and Kruskal–Wallis tests were used to calculate the significance of area under the curve (AUC) means and 95% confidence interval in this study (Supp. Figure. S2). A p-value of ≤ 0.05 (two-tail) was considered significant. For the analysis of clinical variables, a non-parametric test (i.e., Mann–Whitney U test) was used for when the independent variable is dichotomous (e.g., gender), and the Spearman's rank correlation test when the independent variable is continuous (e.g., age) [32]. Data were analyzed using Python SciPy package (version 1.12.0) mann–whitney-u and spearman functions. Only significant clinical variables were reported in Table 4. For correlation significance, (r) = 0–0.30 was regarded as negligible or no correlation, 0.3–0.50 as weak, 0.50–0.70 as moderate, 0.70–0.90 as strong, and 0.90–1.00 as very strong correlation [32–34].

### Supplemental material

Supp. Figure. S1 shows in vivo longitudinal bioluminescence imaging of acute and chronic inflammation in the right rear foot pad, but not the left rear foot pad, of mice. Supp. Figure. S2 shows the AUC values and Wilcoxon Scores of IgG and IgM Classified by inflamed vs. control foot in mice with acute and chronic inflammation. Supp Table. S1 shows some selected statisrical parameters of IgG and IgM in mice with acute and chronic inflammation.

## Results and discussion

To date, the size difference between IgM and IgG is a topic that has not been extensively researched in the field of immunology. Herein, we propose that the difference in size may provide an evolutionary advantage for IgM to target inflammation sites during the early days of infections. Our results showed that IgM has a significantly high relative distribution towards inflammation sites due to their physical characteristics, thereby increasing their chance of binding to pathogens. IgM has multipoint binding sites that confer high overall avidity to pathogens at inflammation sites [1–6, 35]. Once the immune system recognizes a pathogen, it engineers IgG with a higher affinity to pathogens than IgM [1–6]. After almost two weeks, smaller-sized IgG can infiltrate via smaller gap junctions in almost all tissues of the body, thereby preventing the spread of pathogens into other healthy sites [1–11]. We confirmed this hypothesis using different mouse models and clinical data of 20 patients diagnosed with Aspergillus infection.

### Distribution of IgM and IgG in inflammation sites in mice

**Fig. 1:**
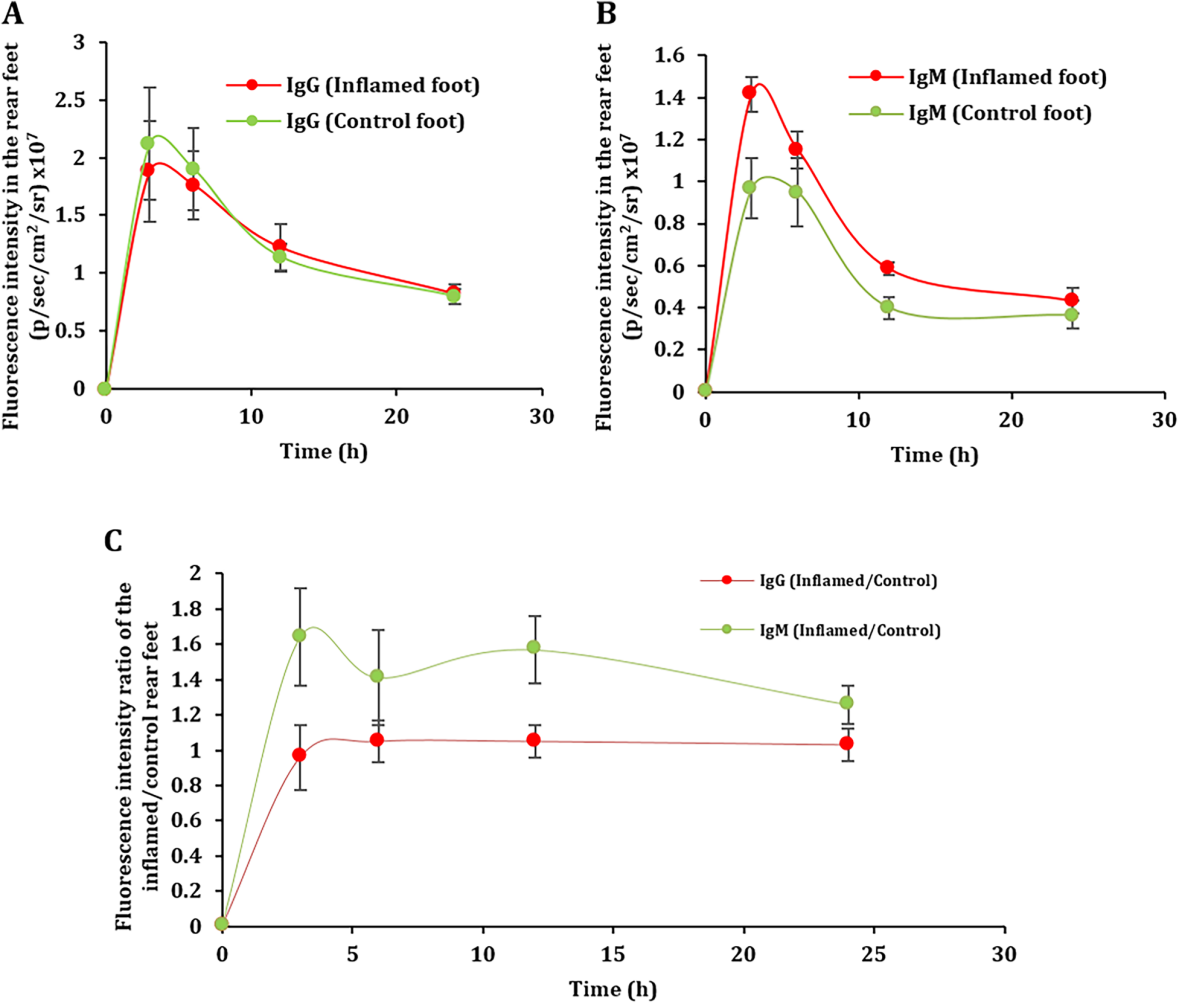
Distribution of IgG and IgM in inflamed foot relative to healthy foot in the same mouse with acute inflammation. **A** IgG fluorescence intensity profiles in the rear feet of the mice. **B** IgM fluorescence intensity profiles in the rear feet of the mice. **C** The ratio of IgG and IgM fluorescence intensity profiles in the inflamed/healthy feet of the mice. Data are mean ± S.E. (n = 6).

IgG and IgM are biologic molecules with particle size in the nanometer scale (i.e., IgG, ~ 5–10 nm; IgM, ~ 50–100 nm) [1–5]. Based on our previous findings with gold nanoparticles and protein-based nanoparticles and other pre-clinical and clinical studies, we hypothesized that the particle size difference between IgG and IgM may affect their distribution and relative accumulation towards inflammation sites [10–18]. First, we tested this hypothesis using the mouse model of LPS-induced acute inflammation in one of the rear footpads (Supp. Figure. S1) by i.v. injecting mice with fluorescently labeled non-binding IgG or IgM. Shown in Fig. 1A and B are the fluorescence intensity vs. time curves in the rear feet, one healthy and another with LPS-induced acute inflammation, of the mice i.v. injected with IgG (Fig. 1A) or IgM (Fig. 1B). Shown in Fig. 1C is the ratio of the fluorescence intensities in the inflamed rear foot vs. the healthy foot as a function of time after mice were i.v. injected with fluorescently labeled IgG or IgM. For IgG, the ratio stayed at 1, indicating that the IgG was distributed to the inflamed and healthy footpads equally. For IgM, the ratio was significantly higher than 1 (p < 0.002), i.e., about 1.6 at 3 h after injection and remained about 1.2 even 24 h after the injection. Clearly, the IgM showed a higher relative distribution than IgG towards inflamed feet, which affected the pharmacokinetic (PK) parameters of IgM in the inflamed foot vs. the healthy foot shown in (Table 1). IgM showed a significant increase of about 23% for the AUC in the inflamed foot vs. healthy foot, compared to a non-significant increase of only 1% for IgG in the same of acute inflammation (Table 1).

**Table 1:** Selected pharmacokinetic parameters of IgG and IgM within the same mouse with acute inflammation.

PK parameter	IgG Inflamed foot	IgG Control foot	IgM Inflamed foot	IgM Control foot
AUC_0-24_ (fluor/ml*h)	29.4 ± 2.2	29.1 ± 2.4	17.9 ± 0.5	13.8 ± 1.3
AUC_0-24_ P-value	P ≤ 0.917		P ≤ 0.037	
Kruskal-Wallis (Pr > ChiSq)				
95% CI AUC _0-24_ (fluor/ml*h)	23.2 to 25.6	22.4 to 25.8	16.5 to 19.3	10.5 to 17.2
Tmax (h)	3	3	3	3
Cl__obs_ (fluor)/(fluor/ml)	2	2.1	2.7	3.4
Cmax (fluor/ml)	1.88	2.12	1.42	0.96

IgG and IgM fluorescence intensity profile with the selected pharmacokinetic parameters in the rear feet of the mice with acute inflammation (n = 6).

**Fig. 2:**
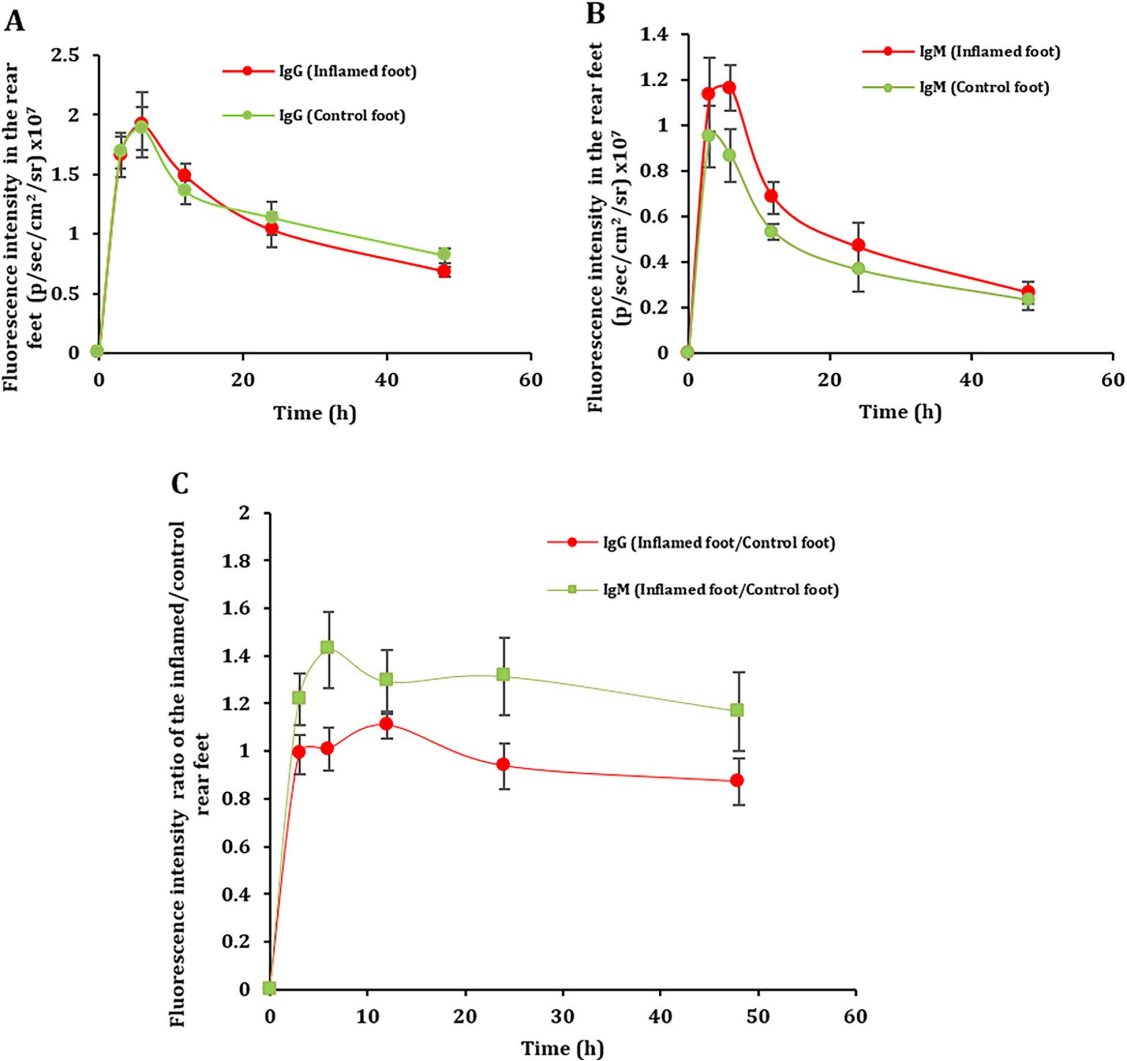
Distribution of IgG and IgM in inflamed foot relative to healthy foot in the same mouse with chronic inflammation. **A** IgG fluorescence intensity profiles in the rear feet of the mice. **B** IgM fluorescence intensity profiles in the rear feet of the mice. **C** IgG and IgM fluorescence intensity profiles ratio of inflamed/healthy feet. Data are mean ± S.E. (n = 6).

Based on the findings above, we hypothesized that the same trend may be observed in chronic inflammation sites. Therefore, we investigated the relative distribution of the IgG and IgM in chronic inflammation sites in mice with LPS-induced chronic inflammation in one of their rear footpads. Mice were i.v. injected with fluorescently labeled IgG or IgM, and shown in Fig. 2 are the fluorescence intensities in the inflamed and healthy rear footpads of the mice. Again, in mice i.v. injected with IgG, there was no apparent difference in the fluorescence intensities in the inflamed and the healthy rear footpads as a function of time (Fig. 2A), but in mice i.v. injected with fluorescently labeled IgM, the fluorescence intensity values tended to be higher in the inflamed footpad than in the healthy control footpad (Fig. 2B). The difference in the kinetics of the IgG and IgM in the inflamed vs. healthy footpads is clearly shown with the ratios of fluorescence intensity in the inflamed foot/the healthy foot as a function of time (Fig. 2C). Shown in Table 2 are selected PK parameters of the IgG and IgM in the rear footpads of the mice, one healthy and another with LPS-induced chronic inflammation. Similar to the acute inflammation, the AUC value of IgM was significantly higher in the inflamed foot of chronic inflammation by 24% than in the control foot. Overall, it appeared that IgM, but not IgG, showed higher distribution in the footpad with chronic inflammation. Our data are in agreement with Subramanian et al.’s finding that IgM antibodies have a higher relative distribution than IgG in *E. coli* infected sites, which we showed is true in chronic inflammation sites as well [6].

**Table 2:** Selected pharmacokinetic parameters of IgG and IgM within the same mouse with chronic inflammation.

PK parameter	IgG Inflamed foot	IgG Control foot	IgM Inflamed foot	IgM Control foot
AUC_0-24_ (fluor/ml*h)	30.1 ± 1.1	29.3 ± 0.9	15.3 ± 0.5	11.6 ± 0.5
AUC_0-24_ P-value	P ≤ 0.749		P ≤ 0.004	
Kruskal-Wallis (Pr > ChiSq)				
95% CI AUC_0-24_ (fluor/ml*h)	27.2 to 33.0	27.0 to 31.5	14.1 to 16.4	10.4 to 12.9
Tmax (h)	6	6	6	6
Cl__obs_ (fluor)/(fluor/ml)	1.2	0.9	1.9	2.2
Cmax (fluor/ml)	1.9	1.9	1.2	0.9

IgG and IgM fluorescence intensity profile with the selected pharmacokinetic parameters in the rear feet of the mice with chronic inflammation (n = 6).

### Distribution of IgM and IgG in tumors in mice

**Fig. 3:**
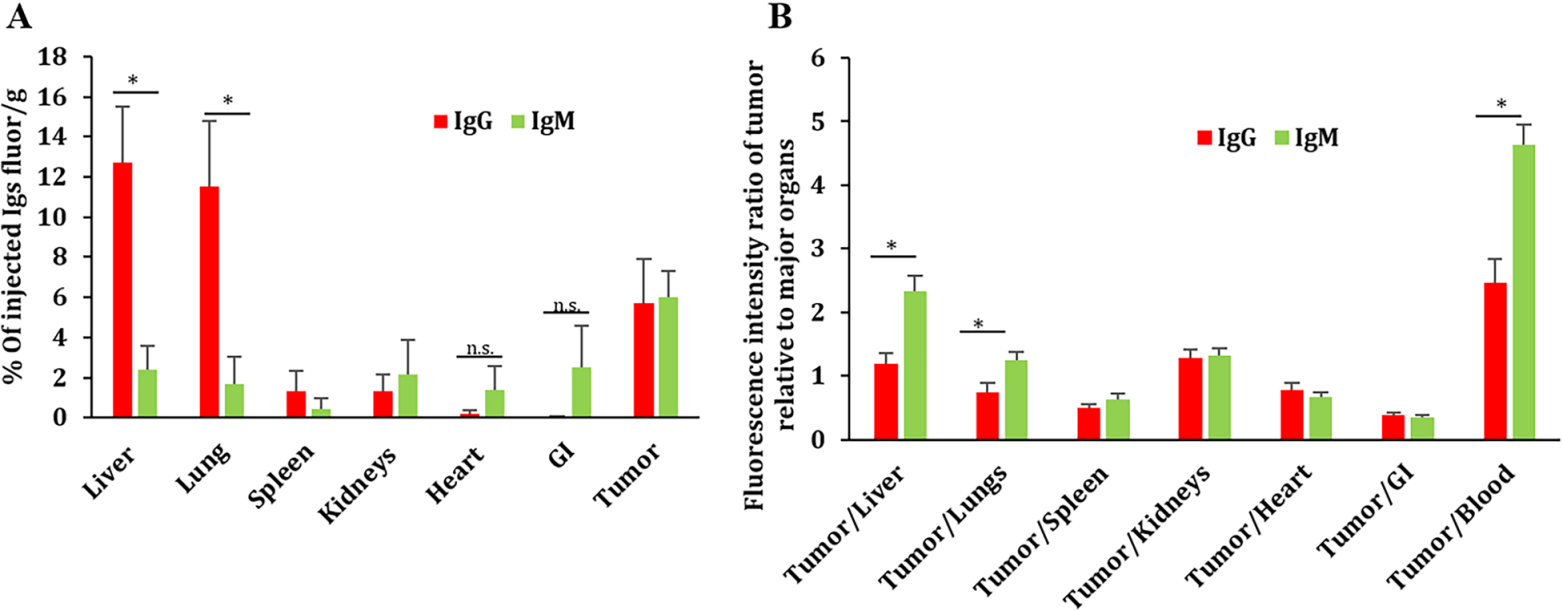
IgG and IgM distribution in mice with M-Wnt tumors. **A** Percent of IgG or IgM detected in tumors, blood, and major organs 24 h after i.v. injection in M-Wnt tumor bearing mice. Shown are the percent of dosed fluorescence intensity normalized to the weight of organs (GI=Gastrointestinal tract) and tumors. Values were after subtracting the mean values from the PBS group. **B** Ratios of IgG and IgM in tumor/organs. Data are mean ± S.D. (n = 3). *p < 0.05 from the respective control. The notation (n.s.) stands for not significantly different (p > 0.05).

Clinical studies have already shown that IgM has a higher relative accumulation than IgG in mammary tumor tissues when compared to non-tumoral mammary tissues [6, 7]. Similarly, several studies suggested that the high avidity of IgM towards apophatic cells is possibly the reason for the higher selective accumulation of IgM in tumors and inflammation sites [6, 7]. However, differences in particle size between IgM and IgG may be also a contributing factor to the difference in IgM/IgG relative distribution towards tumor tissues. To study the distribution of IgG and IgM in tumor tissues, relative to other key organs, we injected (i.v.) M-Wnt tumor-bearing mice with fluorescently labeled IgG or IgM. Shown in Fig. 3A are the percentages of injected IgG and IgM that were detected in tumors and key organs, 24 h after the injection. IgM levels were significantly lower than IgG in the liver and lung (Fig. 3A), likely due to higher blood flow to the liver and lung when compared to other organs [36]. In Fig. 3B, IgM showed a higher tumor to blood, tumor to liver, and tumor to lung ratios than IgG, indicating that IgM had more relative distribution to tumors than IgG. This is probably due to the enhanced permeability and retention (EPR), which is known to enhance the accumulation of larger particles in tumors relative to other healthy tissues [11–21]. Additionally, the much longer half-life for IgGs in the blood (6–8 days) vs. (2 days) for IgMs may be another contributing factor for the differences seen in the blood, liver and lung [37].

**Fig. 4:**
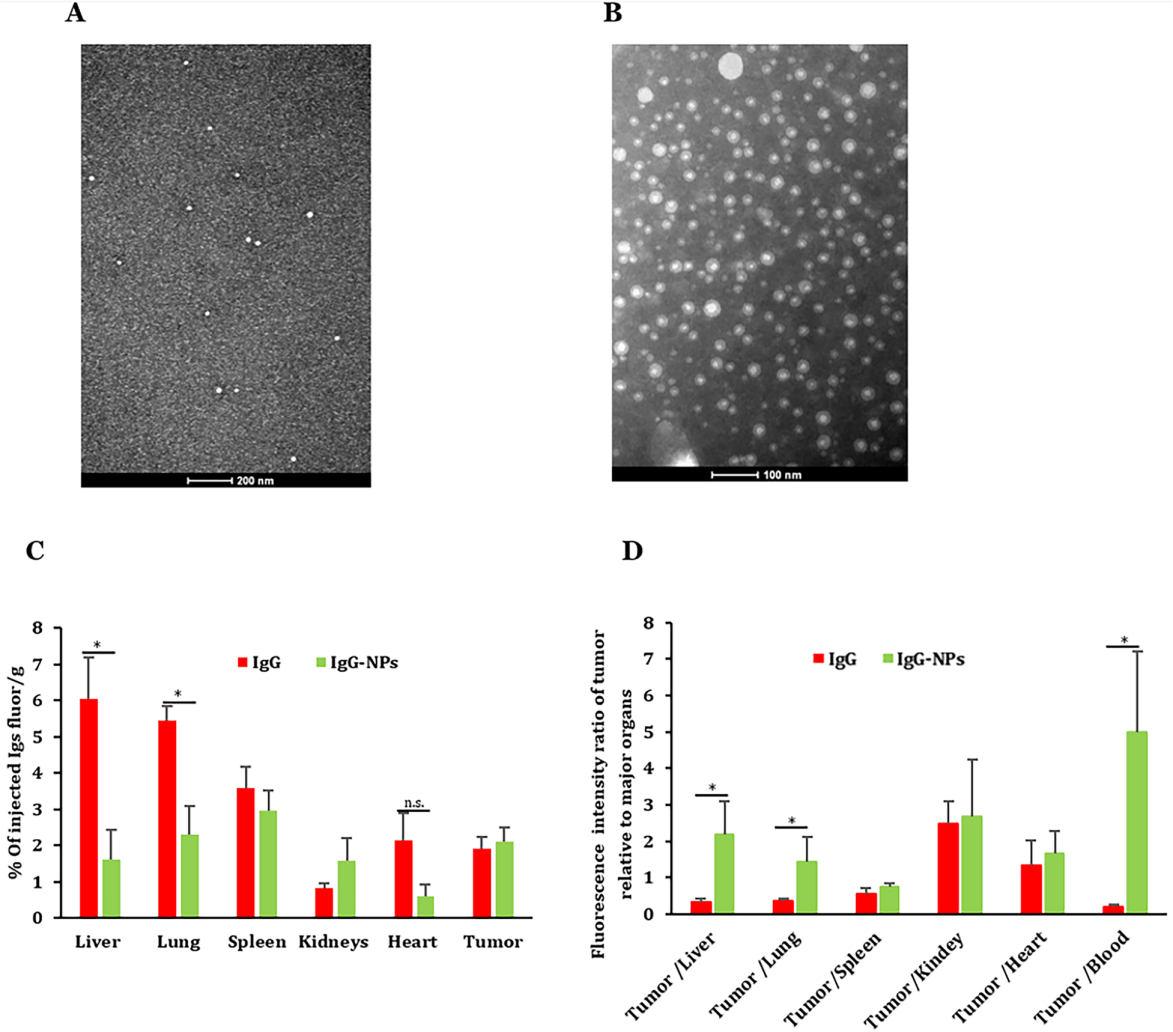
Distribution IgG, free or in IgG-NPs, in mice with M-Wnt tumors. **A** A representative TEM image of IgGs and **B** IgG-NPs. **C** Percent of IgG detected in tumors, blood, and key organs in M-Wnt tumor-bearing mice 24 h after i.v. injection with IgG, free or in IgG-NPs. Shown are the percent of dosed fluorescence intensity normalized to the weight of organs and tumors. **D** Fluorescence intensity ratio of tumor relative to major organs after subtracting the mean values from the PBS group. Data are mean ± S.D. (n = 3). *p < 0.05 from the respective control. The notation (n.s.) stands for not significantly different (p > 0.05).

To further confirm the higher relative distribution of IgM in tumors was size dependent, we prepared IgG nanoparticles (IgG-NPs) following our previous method (Fig. 4A, B) [14]. The IgG and IgG-NPs were labeled fluorescently and i.v. injected into mice with pre-established M-Wnt tumors. Shown in Fig. 4C are the distribution of the IgG and IgG-NPs in tumor tissues and key organs, and the ratios of the fluorescence intensities in tumor tissue vs. organs or blood are shown in Fig. 4D. Overall, the distribution pattern of the IgG-NPs was similar to that of the IgM (Figs. 3A, B vs 4C, D), indicating that difference in the distribution of the IgM and IgG in tumor-bearing mice was related to the physically larger particle size of the IgM.

Similar to the IgM vs. IgG tumor distribution data, IgG-NPs have significantly reduced IgG distribution and accumulation in liver and lung tissues compared to when given as free IgG (Fig. 4C). Moreover, IgG-NPs showed a higher tumor-to-blood ratio than free IgG, indicating that formulating IgG into IgG-NPs may increase its specific distribution to tumors (Fig. 4D). The outcomes confirm that by only modifying the size of IgGs to a size range similar to IgM molecules (Fig. 4A, B), they could change their distribution and relative distributions to tumors and healthy tissues (Fig. 4C, D).

### Relative distribution of IgM and IgG in the plasma and BALF samples of patients diagnosed with *Aspergillus* infection in the lung

*Aspergillus* infection is a fungal disease that can lead to various clinical presentation, including invasive pneumonia [38–41]. Patients with *Aspergillus* infection may exhibit an immune response characterized by an increase in both IgG and IgM antibodies [37–40]. This immune response is crucial for diagnosing and monitoring the progression of *Aspergillus* infection and guiding appropriate treatment interventions [37–40]. The presence of IgG and IgM antibodies in response to *Aspergillus* infection provides valuable clinical data that can aid in assessing the severity of lung inflammation and guiding the effectiveness of therapeutic interventions [37–40]. The important clinical characteristics of the 20 patients diagnosed with *Aspergillus* are presented in Table 3. All patients have an underlying disease (i.e., cancer or organ transplant), which requires the administration of immunosuppressant medications. It has been reported that immunosuppressants used in cancer treatment and organ transplantation are strongly associated with *Aspergillus* infections [37–41].

**Table 3:** Clinical data, medical procedures, and pathological tests of patients diagnosed with Aspergillus.

Feature	(n = 20)
Age (mean, ±S.D.)	43.1, ± 16.86
Sex (male, female)	14, 6
Malignancy, count (DLBCL, Breast Cancer, AML, Myeloid neoplasm, ALL, MDS, NA)	6, 1, 8, 1, 2, 1, 1
Nodules, percentage	75%
Consolidation, percentage	15%
PE, percentage	10%
GGO, percentage	50%
CL, percentage	10%
BAL culture_result, percentage, (*Candida glabrata*, MRSA, Normal flora, *Candida albicans*, *Mycobacterium abscessus*, *Haemophilus influenzae*, *Candida tropicalis*, no organisms)	(5%, 5%, 15%, 5%, 5%, 5%, 65%)
Symptomatic, excluding chronic cough (n=17), percentage	76%
Hypoxia, percentage	10%
ESR (mean, ±SD), excluding pts with NA values (n = 9)	64.44, ± 35.84
CRP (mean, ±SD), excluding pts with NA values (n = 14)	102.64, ± 89.22
RM, percentage, (RSV, Rhinovirus, SARS-CoV-2, Negative, NA)	5%, 15%, 10%, 50%, 20%

All patients have a serious secondary indication requiring immunosuppressant medication administration. *CLD: Chronic Lung Disease , *PE:

Pleural Effusion , *GGO: Ground Glass Opacity, *CL: Cavitary Lesion ,*ESR: Erythrocyte Sedimentation Rate , *CRP: C-Reactive Protein, * DLBCL: diffuse large B cell lymphoma, *AML: Acute Myeloid Leukemia, *ALL: Acute Lymphocytic Leukemia, *MDS: Myelodysplastics Syndromes, *NA: Not Available.

**Fig. 5:**
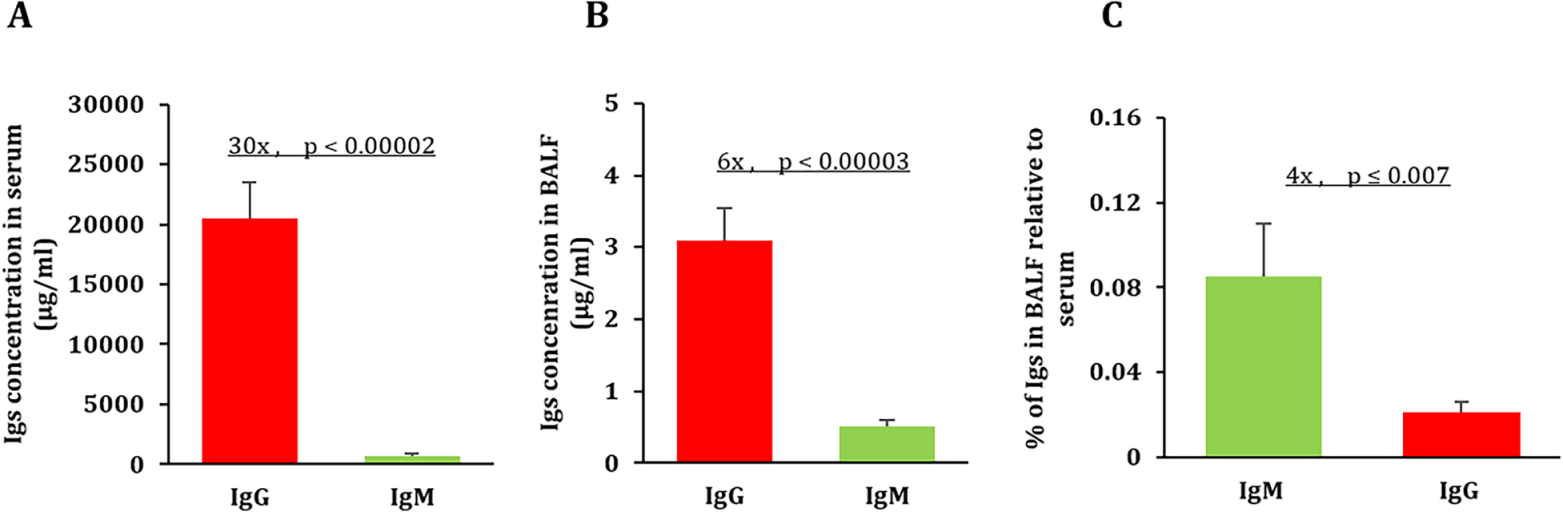
IgG and IgM levels in the serum and BALF fluid from patients diagnosed with Aspergillus. **A** Total IgG and IgM concentrations in the serum were measured by ELISA. **B** Total IgG and IgM concentrations in the BALF fluid were measured by ELISA. **C** The relative IgG and IgM concentrations in the BALF fluid compared to the serum, subjects with a serum IgM value of zero were excluded. Data are mean ± S.E. (n = 20).

As expected, the total human IgG levels were higher than IgM in plasma and BALF samples from patients diagnosed with *Aspergillus* (Fig. 5). In the plasma, IgG concentration was significantly higher than IgM concentration by a factor of 30 (Fig. 5A). However, in BALF samples from the inflamed lungs, IgG was higher than IgM only by a factor of 6 (Fig. 5B). This indicates that IgM has a relatively higher concentration in the BALF samples than IgG, when compared to in plasma samples (Fig. 5C). The total IgG and IgM plasma concentrations reported in this study is slightly higher than normal range in healthy individuals [42]. The IgM concentration in the BALF of healthy individuals is about 0.22 ± 0.03 µg/ml, whereas in asthmatic patients it can range from 0.3 to 1.99 µg/ml [43]. The average concentration of IgM in the BALF samples from the patient diagnosed with *Aspergillus* was about 0.5 ± 0.08 µg/ml (Fig. 5B), which is more than twice the concentration reported in the BALF of healthy individuals [42]. However, the average concentration of IgG (3.1 ± 0.44 µg/ml) in the BALF was lower than average IgG levels reported in healthy individuals which is about 7–9 µg/ml [43]. In other words, in healthy individuals, the ratios of IgG to IgM range between 32 and 41. The observed decrease of the ratio of IgG to IgM in the BALF samples in patients diagnosed with *Aspergillus* could be due to the previously reported effect of immunosuppressant treatments such as steroids, which are known to significantly reduce IgG levels in BALF, but not IgM [44], but could also be a result of the leaky pulmonary capillaries allowing more IgM to enter the BALF [24–28, 46]. Lovegrove et al. reported a significant increase of IgM in the BALF due to alveolar-capillary membrane barrier disruption, which are hallmarks of acute lung injury [44, 45].

Table 4 shows the clinical variables that showed some significant correlation with IgG and/or IgM levels in the serum and/or blood. The IgG distribution to the BALF samples vs. serum was significantly less in male when compared to female (p < 0.02). It has been reported that females tend to have higher total IgG levels than males post-infection, which may have contributed to this newly reported finding [44,45,^47^]. Patients with nodule formation in the lung have significantly less IgM levels in their BALF samples. However, this negative correlation did not affect the overall relative distribution of IgMs to the inflamed lung (Table 4). Finally, a strong negative correlation between IgG concentration in the BALF and the erythrocyte sedimentation rate (ESR) values. This is expected as ESR is known to be a strong marker of acute inflammation that peaks at 48–72 h post-infection [48]. Similar to what was reported by Subramanian et al. in an animal study, the overall outcomes of this clinical study confirm that IgM may have a higher relative distribution in inflammation sites than IgG.

**Table 4:** Mann-Whitney U test and correlation between IgG and IgM levels and patient's clinical data*.*

**Groups **	**IgG in serum **	**IgM in serum **	**IgM in BALF**	**IgG in BALF**	**IgG BALF/Ser **	**IgM BALF/Ser **
Mann-Whitney U test for gender						
***U-statistic ***	31.5	33.0	30.5	43.5	**5.0**	9.0
***P-value***	0.24	0.17	0.36	0.93	**0.02**	0.10
Mann-Whitney U test for pulmonary nodules						
***U-statistic ***	12.0	21.0	22.0	**10.0 **	22.0	22.0
***P-value ***	0.21	0.95	0.19	**0.01 **	1.00	1.00
Correlation with ESR						
***Spearman *** ***correlation ***	− 0.50	0.79	**− 0.73 **	− 0.28	− 0.09	− 0.79
***P-value ***	0.39	0.10	**0.02**	0.46	0.87	0.10

Some patient's characteristics and pathological test results showed a significant correlation with IgG and IgM levels in serum or BAL samples. BAL/Ser is the relative distribution of IgG or IgM to the BAL fluid in the inflamed lung when compared to their levels in the serum.

For gender Mann-Whitney U significant test, male encoded as 1 and female as 0  (n = 9–20), (p < 0.05 is considered significant), (r = 0–0.30 is regarded as negligible or no correlation, 0.3–0.50 as weak, 0.50–0.70 as moderate, 0.70–0.90 as strong and 0.90–1.00 as very strong correlation). ESR: Erythrocyte Sedimentation Rate is (n = 9).

Pulmonary and liver adverse events are commonly associated with monoclonal antibodies that have been approved for clinical use, although the mechanisms underlying such adverse effects are generally not known [44,45,^49^–^52^]. For pulmonary adverse effects there are four main categories: interstitial pneumonitis and fibrosis, acute respiratory distress syndrome (ARDS), bronchiolitis obliterans organizing pneumonia (BOOP), and hypersensitivity reactions [49–52]. The liver is an Fc-receptor-rich organ that helps to increase the circulation and retention time of monoclonal antibodies [49–52]. A possible side effect of antibody therapy is the cytokine-release syndrome that may lead to auto-immune complications via interactions with Fc receptors [49–52]. Due to the longer exposure, life-threatening and fatal cytokine release syndrome has been reported with antibody therapies (e.g., Rituximab for treatment of chronic lymphocytic leukemia (CLL) and non-Hodgkin’s lymphomas) [49–52]. The high concentrations of IgG in the lungs and liver are probably related to the high residual plasma concentrations of the IgG in those organs, which may also be related to the adverse events caused by monoclonal antibodies in the lungs and liver [49–52]. It is posited that switching that antibodies from IgG to IgM may help alleviate the adverse events. We then proposed a strategy to enhance the selective distribution of existing IgG-based mAbs therapies such as anti-TNF-α mAbs by transforming them into larger nanoparticles that respond to high redox activity and/or low pH—conditions commonly found in inflammation and tumor sites [14].

However, this study did not account for other immunological mechanisms that could enhance the distribution of IgM in inflammatory sites and tumor tissues. The high avidity of IgM for cells within tumors and inflammatory sites may also contribute to its increased distribution and accumulation in these areas [10–12]. To minimize the influence of other immunological factors, we used non-binding IgM and IgG in all our animal studies. Additionally, a limitation of our findings is that the newly developed IgG-nanoparticle system is specifically designed for mAbs administered via the intravenous route and may not be applicable to nanoparticles and mAbs delivered through other routes.

## Conclusions

Our immune system through its evolutionary development has engineered different types of Igs (i.e. IgG, IgM, IgA, IgE, and IgD) with different biological and physical characteristics. Herein, we showed that the relative distribution of IgMs was significantly higher than IgG in inflammation sites or tumor tissues when compared to blood and healthy tissues. We confirmed that the difference in size between IgM and IgG may have contributed to the differences in relative distribution in inflammation sites and tumors. However, our study did not take into account other immunological mechanisms that could further enhance the distribution of IgM in inflammatory sites and tumor tissues. Understanding the impact of physical differences on the functions and mechanisms of these antibody classes remains an intriguing area for future studies. Such an understanding of our immune system and how its function may help us develop more effective diagnostic tests, vaccines, and therapeutic interventions against inflammatory diseases and cancers.

## Supplementary Information

Additional file 1.

## Data Availability

The datasets generated during and/or analyzed during the current study are available from the corresponding authors upon reasonable request.
