# Supplementary material for: IgM has a better relative distribution in inflammation sites and tumor tissues than IgG
Source: J Nanobiotechnology. 2025 Mar 28;23:253. doi: 10.1186/s12951-025-03213-4 (PMC11954317; doi:10.1186/s12951-025-03213-4)
Supplement: Supplementary file 1 — Additional file 1. [file 12951_2025_3213_MOESM1_ESM.pdf]

IgM has a better relative distribution in inflammation sites and tumor tissues than IgG

Abdulaziz M. Aldayel[1,2,5*], Mohammad Bosaeed[3,4,5], Sarah Almansour[4], Naif Alharbi[3,5], Mohammed Alenazi[3], Haya A. Aljami[3] , Omar Aldibasi[5,6], Abdulrhman Aljouie[5,6], Haiyue Xu[1] and Zhengrong Cui[1*]

[1] The University of Texas at Austin, College of Pharmacy, Division of Molecular Pharmaceutics and Drug Delivery, Austin, TX 78712, United States.

[2] Nanomedicine Department, King Abdullah International Medical Research Center (KAIMRC). King Abdulaziz Medical City (KAMC), Riyadh, 11426, Saudi Arabia.

[3] Infectious Diseases Research Department, King Abdullah International Medical Research Center. King Abdulaziz Medical City (KAMC), Riyadh, 11426, Saudi Arabia.

[4] Infectious Diseases Department, King Fahad Hospital (KFH), King Abdulaziz Medical City (KAMC), Riyadh, 11426, Saudi Arabia.

[5]Department of Biostatistics and Bioinformatics, King Abdullah International Medical Research Center, King Saud bin Abdulaziz University for Health Sciences, Riyadh, Saudi Arabia

[6] King Saud bin Abdulaziz University for Health Sciences (KSAU-HS), King Abdulaziz Medical City (KAMC), Riyadh, 11426, Saudi Arabia.

***Correspondence to:**

Abdulaziz Aldayel, Ph.D.

Email: abdulazizaldayel777@gmail.com

Zhengrong Cui, Ph.D.

Tel: (512) 495-4758

Email: Zhengrong.cui@austin.utexas.edu

Supp. Figure. S1

A

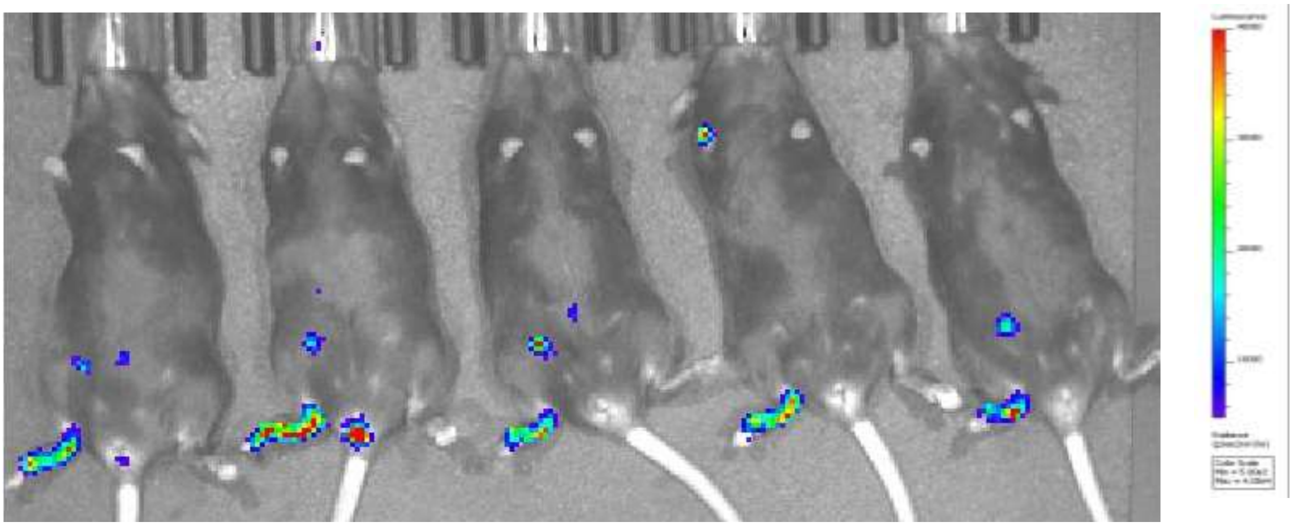

B

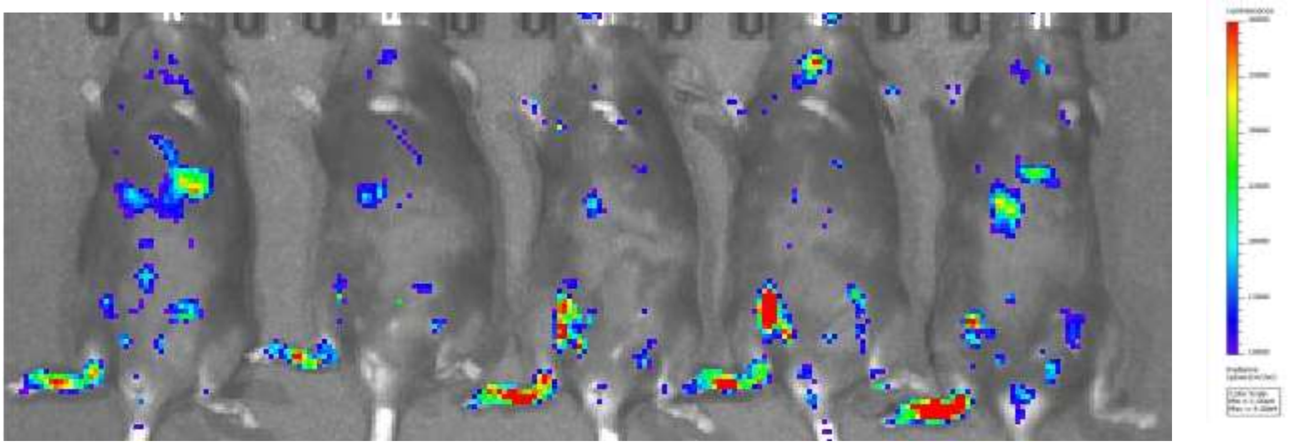

Supp. Figure. S1. ***In vivo* longitudinal bioluminescence imaging of acute and chronic inflammation in the right rear foot pad, but not the left rear foot pad, of mice.** We induced local tissue inflammation by s.c. injection of 50 μg LPS. (A) Mice were given an i.p. injection of luminol (100 mg/kg) and imaged on day 3. (B) Mice were given an i.p. injection of lucigenin (25 mg/kg) and imaged on day 8.

Supp. Table. S1. Selected statisrical parameters of IgG and IgM in mice with acute and chronic inflammation. Tukey-Kramer adjustment was used to calculate the significant distribution of IgG and IgM in inflamed vs. control feet over time. (A) IgG and (B) IgM distribution to mice feet within acute inflammation. (C) IgG and (D) IgM distribution to mice feet within chronic inflammation. (n=5-6)

A

Type 3 tests of fixed effects IgG acute inflammation				
Effect	Num DF	Den DF	F Value	Pr > F
Time	3	12	5.86	0.0106
Tissue	1	4	0.05	0.8263
Time*Tissue	3	12	0.12	0.9467
Inflamed vs. Control, Tukey-Kramer , adjusted P ≤ 0.8263				

B

Type 3 tests of fixed effects IgM acute inflammation				
Effect	Num DF	Den DF	F Value	Pr > F
Time	3	15	18.46	<.0001
Tissue	1	5	13.03	0.0154
Time*Tissue	3	15	1.79	0.1931
Inflamed vs. Control, Tukey-Kramer , adjusted P ≤ 0.0154				

C

Type 3 tests of fixed effects IgG chronic inflammation				
Effect	Num DF	Den DF	F Value	Pr > F
Time	3	30	8.34	0.0003
Tissue	1	10	0.08	0.7857
Time*Tissue	3	30	0.26	0.8522
Inflamed vs. Control, Tukey-Kramer , adjusted P ≤ 0.7857				

D

Type 3 tests of fixed effects IgM chronic inflammation				
Effect	Num DF	Den DF	F Value	Pr > F
Time	3	30	14.44	<.0001
Tissue	1	10	18.82	0.0015
Time*Tissue	3	30	0.33	0.8049
Inflamed vs. Control, Tukey-Kramer , adjusted P ≤ 0.0015				

Supp. Figure. S2

A

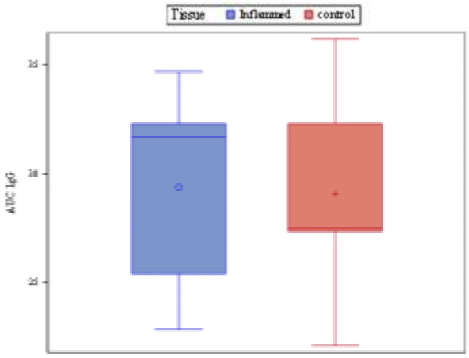

B

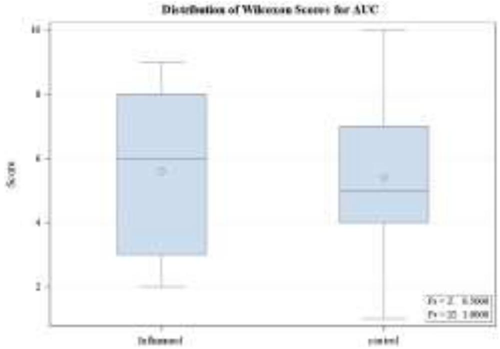

C

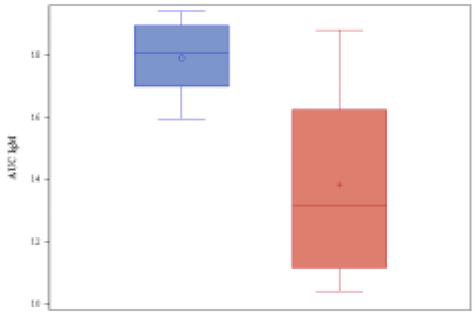

D

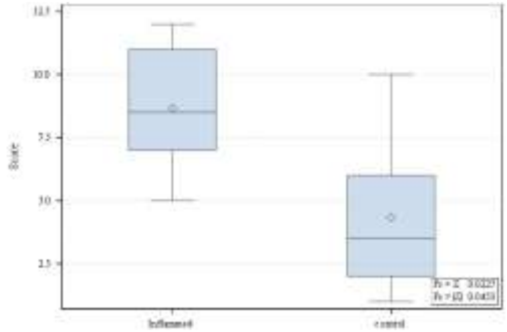

E

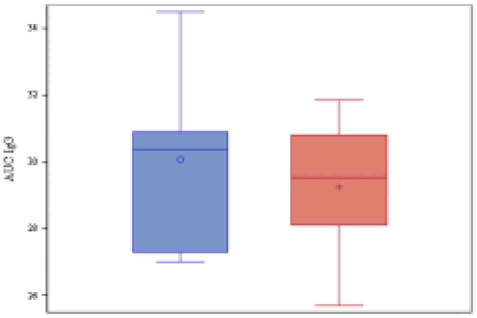

F

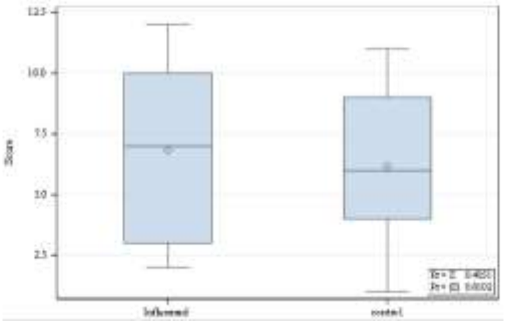

G

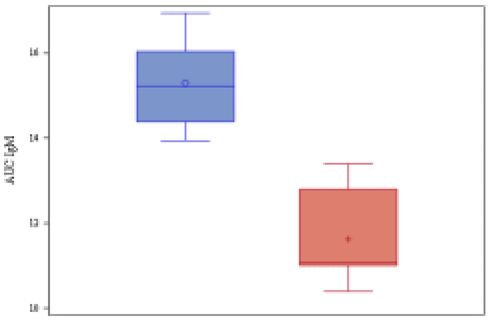

H

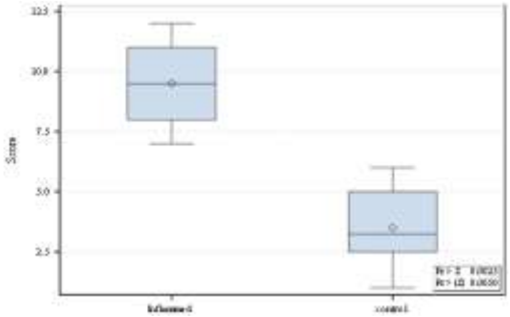

Supp. Figure. S2. **Area under the curve (AUC) values and Wilcoxon Scores of IgG and IgM Classified by inflamed vs. control foot in mice with acute and chronic inflammation.** (A) AUC and (B) Wilcoxon Scores IgG distribution to mice feet within acute inflammation, (C) AUC and (D) Wilcoxon Scores IgM distribution to mice feet within acute inflammation. (E) AUC and (F) Wilcoxon Scores IgG distribution to mice feet within chronic inflammation, (g) AUC and (h) Wilcoxon Scores IgM distribution to mice feet within chronic inflammation. Data are mean ± S.E.M. (n=5-6).
